# Non-pharmacological interventions for patients with dementia: A protocol for a systematic review and meta-analysis

**DOI:** 10.1097/MD.0000000000017279

**Published:** 2019-09-20

**Authors:** Go Eun Lee, Ju Yeon Kim, Jin Hyeong Jung, Hyung won Kang, In Chul Jung

**Affiliations:** a Department of Korean Neuropsychiatry Medicine, Wonkwang University Sanbon Hospital, Gyeonggi-do; b Department of Neuropsychiatry of Korean Medicine, Dunsan Korean Medicine Hospital of Daejeon University, Daejeon, Republic of Korea.

**Keywords:** dementia, non-pharmacological intervention, protocol, systematic review

## Abstract

**Background:**

This protocol for a systematic review describes the methods that will be used to evaluate the efficacy and safety of non-pharmacological interventions for patients with dementia.

**Methods:**

We will search ALOIS, the specialized register of the Cochrane Dementia and Cognitive Improvement Group (CDCIG), without language or publication status restrictions. Additional separate searches will be run in many of the above six databases to ensure the most up-to-date results are retrieved.

The study selection and data extraction will be performed independently by two authors and only randomized controlled trials will be included. The risk of bias will be assessed independently by two authors following the Cochrane Handbook for Systematic Reviews of Interventions. We will use RevMan software and random-effects models to assess the heterogeneity and data synthesis.

If any plan for documenting important protocol amendments changes, the researchers will make a revision agreement and then register the modification on PROSPERO.

**Conclusion:**

Through this systematic review, a comprehensive understanding of current non-pharmacological interventions on dementia will be available. Meanwhile, it will provide basic evidence for further clinical research.

**Ethics and dissemination:**

Ethical approval is not required because no individual patient's data are included in this paper. This study will be disseminated through conference presentation.

**Prospero registration number:**

CRD42019136435

## 1 Introduction

Dementia is a syndrome characterized by cognitive impairment and high frequencies of concurrent neuropsychiatric symptoms such as agitation, aggression, and apathy. In this regard, dementia contributes to the impairment in ADL, caregiver burden, and high levels of healthcare utilization and costs.^[1,2]^

About 47 million people were affected by dementia worldwide in 2016.^[3]^ In a recent systematic review, among individuals over 60 in the community, the annual prevalence and incidence of dementia were estimated at 69.07 (CI 95%: 52.36–91.11) and 17.18 (CI95%; 13.90–21.23)^[4]^ per 1000 persons, respectively. Aging was associated with higher dementia prevalence and incidence.^[4]^ Moreover, it is estimated that more than 131 million people will be affected by dementia in 2050.^[3]^

Common types of dementia include Alzheimer's disease (AD), vascular dementia (VD), dementia with Lewy bodies (DLB), dementia in Parkinson's disease (PDD) and frontotemporal dementia (FTD). The boundaries between the different subtypes are indistinct and multiple types often coexist in at least 25% of people with dementia.^[1,5]^

There is no currently available cure for dementia. Nevertheless, various treatments and interventions have been researched. Despite certain limitations, medications are considered the primary treatment.^[6]^ Licensed medications are only available for AD and PDD, which have been shown to slow the cognitive decline by up to one year but not modify the disease's course.^[7]^ Additionally, antipsychotic drugs are widely used for behavioral and psychological symptoms (BPSD), which approximately 50% of patient's experience. However, antipsychotic drugs have serious side effects on the elderly, increasing their risk of cerebrovascular events and even death.^[8–10]^

In addition to medication therapies for dementia, several non-pharmacological interventions have been proposed because they carry fewer risks and adverse effects.^[11]^ In particular, non-pharmacological interventions are recommended as the preferred first-line treatment for BPSD.^[12–14]^ Non-pharmacological interventions can also postpone the institutionalization of patients to reduce the burden on caregivers.^[15]^ Therefore, non-pharmacological approaches are receiving increased attention as a critical part of dementia care.^[13]^

Various non-pharmacological interventions have been applied to people with dementia and their caregivers. There is various evidence that exercise programs such as aerobic exercise and strength training,^[16–19]^ occupational therapies that involve ADL training and environmental adaptations have positive effects on patients’ daily function.^[18,20,21]^ Inconsistent evidence has been reported^[21–24]^ regarding cognitive training, cognitive stimulation, and cognitive rehabilitation (group or individual). Meanwhile, other modalities such as art therapy and music therapy still lack evidence.^[25,26]^

For non-pharmacological interventions, most existing reviews have analyzed a specific type of dementia or participants in homes or community settings.^[17,21,27]^ Some systematic reviews have investigated a specific symptom of BPSD such as agitation,^[28]^ sleep disturbance,^[29]^ wandering,^[30]^ otherwise dealing with a specific kind of intervention alone (e.g., light therapy,^[31]^ snoezelen,^[32]^ validation therapy,^[33]^ aromatherapy,^[34]^ homeopathy,^[35]^ reminiscence therapy,^[36]^ stimulated presence therapy^[37]^). In another case, a review focused on various kinds of non-pharmacological intervention but only investigated BPSD,^[38]^ ignoring other signs of effectiveness such as cognition, functional level, and caregiver burden.

Therefore, this systematic review aims to investigate any kind of non-pharmacological intervention, regardless of setting and type of dementia, unlike previous reviews. This review will provide valuable evidence to identify effective intervention approaches and develop new interventions.

## 2 Methods

This systematic review protocol has been registered on the international prospective register of systematic review (PROSPERO) and its registration number is CRD42019136435. The protocol will be strictly developed under the guidelines of Preferred Reporting Items for Systematic Reviews and Meta-analyses Protocols (PRISMA-P).^[39]^

### 2.1 Eligible criteria for study selection

#### 2.1.1 Types of studies

We will only include randomized controlled trials (RCTs) that investigate the effects of non-pharmacological interventions to improve any kinds of symptoms in people with dementia. In addition, cross-over designs will be included. Studies without sufficient description of the randomization process will be excluded.

#### 2.1.2 Types of participants

Participants diagnosed with dementia using any kind of diagnostic criteria will be included, irrespective of age, gender, nationality, type of dementia, or severity of cognitive impairment.

#### 2.1.3 Types of interventions

##### 2.1.3.1 Experimental interventions

Any kind of non-pharmacological intervention will be included and studies that used medication alongside non-pharmacological intervention will also be included.

##### 2.1.3.2 Control interventions

Usual care, medication, or no treatment will be considered as control interventions. Additionally, studies using any other non-pharmacological intervention in the control group will be included.

#### 2.1.4 Types of outcome measures

Short-term (up to 6 weeks) and long-term (over 6 weeks) outcomes will be investigated separately if possible.

##### 2.1.4.1 Primary outcomes

The primary outcome measures are as follows:

(1) Global or specific cognitive function measured by validated scales
(2) Functional performance in activities of daily living (ADL)
(3) Behavioral and psychological symptoms of dementia (BPSD) measured by validated scales (e.g., the Neuropsychiatric Inventory (NPI)^[40]^)
(4) Overall dementia severity measured by validated tools (e.g., Clinical dementia rating scale (CDR)^[41]^)
(5) Adverse events

##### 2.1.4.2 Secondary outcomes

The secondary outcome measures are as follows:

(1) Quality of life for people with dementia
(2) Distress or quality of life of caregivers
(3) Institutionalization
(4) Compliance with the intervention

### 2.2 Search methods for the identification of studies

#### 2.2.1 Electronic searches

We will search ALOIS, the comprehensive register of trials of the Cochrane Dementia and Cognitive Improvement Group (CDCIG). ALOIS contains dementia and cognitive improvement studies identified from:

1. Monthly searches of multiple major healthcare databases: MEDLINE, EMBASE, CINAHL, PsychINFO, and Lilacs;
2. Monthly searches of multiple trial registers: metaRegister of Controlled Trials; UMIN Japan Trial Register; the World Health Organization (WHO) portal (covers ClinicalTRials.gov; International Standard Randomized Controlled Trial Number (ISRCTN); the Chinese Clinical Trials Register; the German Clinical Trials Register; the Iranian Registry of Clinical Trials, and the Netherlands National Trials Register, plus others)
3. Quarterly search of the Cochrane Library's Central Register of Controlled Trials (CENTRAL)
4. Biannual searches of several grey literature sources: ISI Web of Knowledge Conference Proceedings; Index to Theses; Australasian Digital Theses.

To view a list of all sources searched for ALOIS see the ALOIS website (www.medicine.ox.ad.uk.alois).

We will carry out additional searches in many of the ALOIS information sources to ensure that the most up-to-date results are retrieved.

Table 1 shows the search strategies for MEDLINE.

**Table 1:**
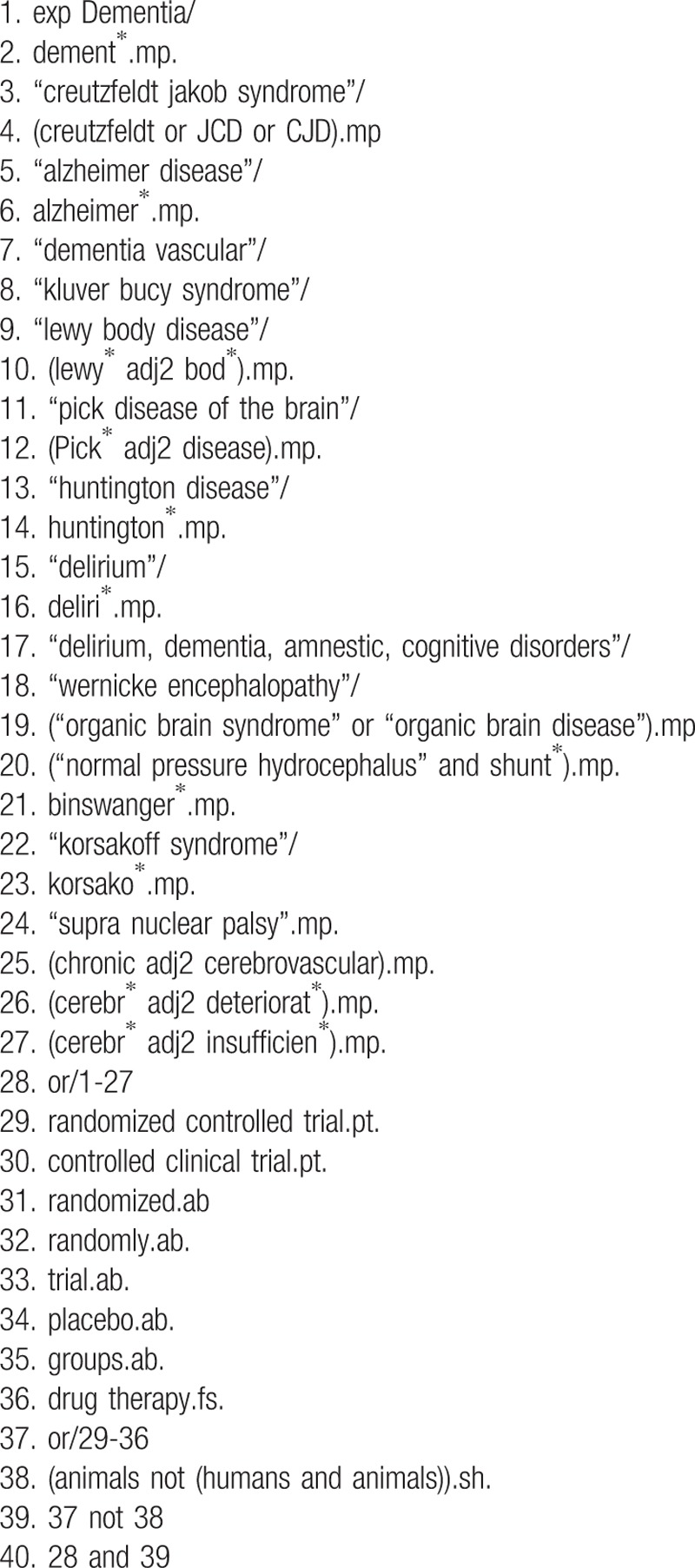
Search Strategies for MEDLINE and EMBASE.

#### 2.2.2 Searching other resources

We will review the reference lists and citations of the included studies, relevant reviews, and trials identified through this search.

### 2.3 Data collection and analysis

#### 2.3.1 Study selection

The study selection will be conducted by 2 review authors independently assessing titles and abstracts after removing duplicates from all search results. Then, full texts will be obtained and assessed for inclusion and exclusion criteria. If no consensus is reached, we will try to resolve disagreements on the eligibility of studies through discussion or—if required—asking a third experienced review author.

#### 2.3.2 Data extraction and management

Two authors will read and extract the data from studies independently. Disagreements will be resolved through discussion with a third review author to reach consensus. We will use a standardized data extraction form that includes the source, first and corresponding author, year of publication, country, trial designation, characteristics of participants, interventions, comparators, duration, outcomes, and adverse events according to the Cochrane Handbook for Systematic Reviews of Interventions (Chapter 7.3).^[42]^

#### 2.3.3 Assessment of bias risk and quality of included studies

We will follow the Cochrane Handbook for Systematic Reviews of Interventions to assess the risk of bias among the included studies.^[42]^ Two independent authors will evaluate the methodological quality of the included studies to identify any potential bias risk. This risk of bias tool examines 6 domains (sequence generation, allocation concealment, blinding, incomplete outcome data, selective outcome reporting, and other sources of bias). Each domain will be assessed as having either a low or high risk of bias. When the details reported in the study are insufficient to evaluate the risk, it will be classified as unclear.

#### 2.3.4 Measurements of treatment effect

The statistical analyses will be performed using Review Manager Version 5.3. For continuous data, mean difference (MD) or standardized mean difference (SMD) with 95% confidence intervals (CIs) will be used. For the analysis of dichotomous outcomes, data risk ratios (RR) with 95% CIs will be calculated.

#### 2.3.5 Dealing with missing data

Missing data will be thoroughly reported. If possible, we will contact the authors and request additional information about missing data via email. If we are unable to retrieve complete data, we will report this in the assessment of bias risk and the impact of missing data will be considered in the data analysis. We will carry out analyses on intention-to-treat (ITT) where available. If the included studies do not provide ITT data, we will use completer-only data.

#### 2.3.6 Assessment of heterogeneity

The statistical heterogeneity will be assessed by I-squared statistic and visual inspections of forest plots using RevMan software;^[43]^ we will consider I-squared values >75% indicative of high heterogeneity.

#### 2.3.7 Assessment of reporting biases

If the number of trials included in the meta-analysis is >10, we will prepare funnel plots to visually estimate the reporting bias.

#### 2.3.8 Data synthesis

We expect frequent clinical heterogeneity due to the complexity of interventions in the trials. Therefore, only random-effects models will be used in the meta-analyses. When it is believed that the heterogeneity is too high for the results to be synthesized (an I-squared value >75%), subgroup analysis will be considered if possible.

#### 2.3.9 Subgroup analysis

If available, we will conduct the following subgroup analyses:

(1) Type and severity of dementia
(2) Different setting
(3) Type of intervention

#### 2.3.10 Sensitivity analysis

Sensitivity analysis will be performed to evaluate the effects of low-quality studies and outliers.

## 3 Ethics and dissemination

Ethical approval is not required in this protocol because the data used in this systematic review will not be individual patient data; there will be no concerns regarding individual privacy. The results will be disseminated by the publication of a manuscript in a peer-reviewed journal or presentation at a relevant conference.

## 4 Discussion

Dementia is the most common type of neurodegenerative disease. Since dementia is associated with age, the prevalence of dementia is increasing around the world as the average life expectancy is lengthening. In addition, the socioeconomic costs of dementia are increasing annually.^[44]^ Unfortunately, definitive pathophysiology and treatment for dementia have not yet been identified. Clinically used medications merely delay the progression of cognitive decline in patients with dementia. In particular, there have been reports that medications for BPSD have some limitations and serious adverse events in spite of their effectiveness.^[45]^ These limited benefits and potential harms mean that non-pharmacological interventions have recently been considered an essential part in the treatment of dementia in clinical practice. However, the previously reported systematic reviews were limited to specific symptoms of dementia or individual non-pharmacological therapies.

Therefore, we will carry out this systematic review to estimate the more general effectiveness and safety of non-pharmacological interventions on dementia irrespective of the subtype. Furthermore, this systematic review will summarize the current state of each modality of non-pharmacological intervention for dementia.

## Author contributions

**Conceptualization**: Go-Eun Lee and In Chul Jung

**Data curation**: Go-Eun Lee

**Formal analysis**: Go-Eun Lee

**Funding acquisition**: In Chul Jung

**Investigation**: Go-Eun Lee and Jin Hyeong Jung

**Methodology**: Go-Eun Lee and Hyung Won Kang

**Resources**: Go-Eun Lee and Jin Hyeong Jung

**Software**: In Chul Jung

**Validation**: Go-Eun Lee and Ju Yeon Kim

**Visualization**: Go-Eun Lee and Hyung Won Kang

**Writing – original draft**: Go-Eun Lee

**Writing – review & editing**: Go-Eun Lee, Ju Yeon Kim, and In Chul Jung.
